# Eco-Friendly Solvent-Based Fabrication of Single-Layer and Polydopamine-Modified Bilayer PVDF-HFP Membranes

**DOI:** 10.3390/membranes16090300

**Published:** 2026-09-13

**Authors:** Tridwip Sen, Eesh Kulshrestha, Muhammad Usman Yousaf, Minwoo Jung, Tequila A. L. Harris, Isabel C. Escobar

**Affiliations:** 1 Department of Chemical and Materials Engineering, Stanley and Karen Pigman College of Engineering, University of Kentucky, Lexington, KY 40506, USA; 2 George W. Woodruff School of Mechanical Engineering, Georgia Institute of Technology, Atlanta, GA 30332, USA

**Keywords:** bilayer membranes, membrane fabrication, slot-die coating, eco-friendly solvents, scale-up

## Abstract

Polymeric membrane systems have emerged as an effective approach for water separations due to their high separation efficiency, simplicity, and adaptability to a wide range of water treatment applications. However, traditional membrane fabrication processes often rely on toxic organic solvents, such as N-methyl-2-pyrrolidone (NMP) and dimethylacetamide (DMAc), which pose environmental and health risks. Eco-friendly solvents have been investigated as an alternative to traditional toxic solvents. This study investigates the fabrication and performance of polymeric membranes using eco-friendly solvent systems, with a focus on bilayer membranes designed to improve separation performance over traditional single-layer membranes. Membranes were fabricated using eco-friendly solvents, Rhodiasolv© PolarClean and gamma-valerolactone in combination with polymers polysulfone (PSf) and poly(vinylidene fluoride-co-hexafluoropropylene) (PVDF-HFP). Membranes were fabricated using nonsolvent-induced phase separation (NIPS) through doctor blade extrusion (DBE) and slot-die coating (SDC) methods in order to compare traditional laboratory-scale casting methods (DBE) with scalable fabrication techniques (SDC). Bilayer membranes were investigated to address limitations presented by single-layer membranes, with polydopamine (PDA) incorporated as an adhesion-promoting additive. Membrane characterization included scanning electron microscopy (SEM), contact angle measurements, and tensile testing, along with water permeability and solute rejection tests. Results showed that polymer concentration and casting method influenced permeability and solute rejection, with the SDC bilayers reaching the highest initial BSA rejection. Filtration used deionized water and model aqueous feeds containing 100 ppm NaCl, 100 ppm CaCl_2_, and 100 ppm bovine serum albumin (BSA).

## 1. Introduction

Membrane technology has been utilized in liquid and gas separations for decades due to the relative ease of fabrication and operation, high selectivity rates, and absence of sorbent regeneration [1]. Along with their low chemical usage and easy accessibility, membranes can potentially bridge the economic and sustainability gap in a number of fields [2]. In particular, membranes are playing a growing role in desalination, water treatment, and food and pharmaceutical industry applications [2,3,4,5]. In the water treatment sector, membranes are an effective technology for removing pathogenic bacteria, viruses, suspended solids, and other contaminants from drinking water.

The majority of membrane studies have been primarily performed on single polymer membranes, which are referred to here as single-layer membranes used to create a porous structure capable of separations [6]. However, despite their widespread use, single-layer membranes are limited in their performance. Since just one material is required to control the selectivity, permeability, and mechanical stability, optimizing one feature may negatively affect the others. Along with the stability of the membrane, the surface is also affected by the limitations of one material. Modifying the surface chemistry of the membrane can influence the hydrophobicity of the membrane and can impact fouling [7]. Changing the surface of the membrane and how it interacts with feed solutions can unintentionally influence permeability or mechanical strength as well. Altering any aspect of the single-layer membrane causes changes in the other aspects, forcing a balance to be found [8]. In order to combat these limitations that the single-layer membranes present, multilayer membranes have been proposed as a next step [9,10]. Specifically, double-layer membranes, or bilayer membranes, have shown promise in combatting these challenges. A bilayer membrane consists of two distinct polymer layers within the membrane, with each one being tailored for a specific final purpose, such as controlling solute rejection, adding mechanical stability, or influencing selectivity mechanisms. Bilayer membranes allow for improved filtration and rejection outcomes, while allowing for optimization of mechanical and structural support since both layers of the bilayer membrane are independently tunable [10,11].

Historically, the development of bilayer membrane technology began with thin-film composite (TFC) membranes, where a thin selective layer of polymer is deposited on top of a porous support layer. These systems demonstrate that separating the selective aspect from the structural function could improve the membrane permeability while maintaining higher solute rejection levels [12]. This has since evolved into polymeric bilayer membranes, where two polymer layers are cast simultaneously to form a single structure. Examples of this are used in seawater desalination, where bilayer membranes with additives, such as silver nanoparticles, are implemented; they can operate efficiently by rejecting smaller particles while also surviving harsh conditions such as hard metals and acidic mediums [13]. Bilayer membrane research has explored the use of different polymer combinations to optimize membrane performance. One polymer may be selected for its excellent chemical resistance and selective properties, while another may provide enhanced compatibility with support materials [14,15].

While bilayer membranes offer many advantages to single-layer membranes, their fabrication process introduces additional complications. One issue with bilayer membrane fabrication is ensuring adhesion between polymer layers. If the layers cannot bind effectively during casting and phase inversion, the membrane may experience delamination, where the layers separate when exposed to mechanical stress or during filtration [16]. Delamination can be detrimental to membrane performance and structural integrity. Attaining adequate adhesion between polymer layers depends on a variety of factors, such as polymer compatibility, solvent selection, and the kinetics associated with phase inversion. If the polymers used in the two layers have poor chemical compatibility or limited intermolecular interactions, there may not be adequate adhesion in the layers. Typically, additives or adhesion-promoting materials, such as polydopamine (PDA) or poly(methyl methacrylate) (PMMA), are implemented to enhance bonding between layers through intermolecular interactions [17,18].

Another challenge in bilayer membrane fabrication lies in controlling the phase inversion process of both polymer layers simultaneously. When the thin film is immersed in the nonsolvent bath during the NIPS process, both layers undergo demixing beginning at the same time at differing rates. However, if there is a large difference in the rate of demixing of each layer, issues such as structural defects, uneven surfaces, or membrane distortion can occur [19].

A final challenge with bilayer membranes lies in their scalability. While laboratory-scale methods such as doctor blade extrusion (DBE) allow for bilayer membranes to be studied under controlled conditions, scaling these techniques to industrial production requires the development of continuous coating methods for multiple layers, introducing complexities in controlling numerous parameters simultaneously. Slot-die coating and roll-to-roll processing are methods that could be used for this, as they are capable of depositing multiple polymer layers at the same time, while maintaining uniform thickness and speeds [20]. Practical scale-up also requires evaluation of the coating window, bead stability, support wetting, defect formation, solvent recovery, cleaning, production rate, and cost.

In this work, a comparative study was conducted between single-layer and bilayer membranes composed of polysulfone (PSf) and polyvinylidene fluoride-hexafluoropropylene (PVDF-HFP). An additional goal was to fabricate bilayer membranes by doctor blade and slot-die coating using different polymer concentrations, solvents, and an adhesion-promoting additive. The eco-friendly solvents Rhodiasolv© PolarClean (PC) and gamma-valerolactone (GVL) were used for single- and bilayer membrane fabrication. Characterization included tensile testing, water contact-angle measurement, and scanning electron microscopy (SEM). These measurements were used to interpret possible relationships among dope viscosity, polymer concentration, membrane morphology, and phase inversion. Finally, rejection studies evaluated relationships between water permeability and rejection of ions and proteins.

The specific contribution of this work is the combined evaluation of the PolarClean/GVL solvent system, a PDA-containing PVDF-HFP/PSf bilayer architecture, DBE-to-SDC translation, and model-feed membrane performance in one study.

### Literature Context and Study Contribution

The recent literature shows progress in eco-friendly solvent membrane fabrication, bilayer membrane design, and scalable coating; however, these elements are often studied separately. Table 1 summarizes representative studies and the gap addressed here.

## 2. Materials and Methods

### 2.1. Selected Materials

The performance of polymeric thin-film membranes is strongly influenced by the selection of polymers, solvents, potential additives, and other support materials implemented during fabrication. The selection of these components must be done carefully to ensure the formation of stable casting solutions and favorable phase inversion behavior. Within these experiments, the material selection process emphasized balancing membrane performance, potential for scale-up, and environmental sustainability.

#### 2.1.1. Polymer Selection

The selection of the polymer is crucial for membrane fabrication because the chemical structure and physical properties of the polymer determine the performance and morphological characteristics of the resulting membrane. In this work, polyvinylidene fluoride-hexafluoropropylene (PVDF-HFP) was obtained from Arkema (Radnor, PA, USA), and polysulfone (PSf, average Mw ~35,000 g mol^−1^ by LS, Mn ~16,000 g mol^−1^ by MO, pellets) was purchased from Sigma-Aldrich (St. Louis, MO, USA). They were selected as the polymers used in the membrane fabrication processes. Both polymers are widely used in drinking water treatment membranes due to their favorable properties, such as chemical stability and thermal resistance. Even though both polymers are favorable for water separations on their own and can dissolve completely with the selected solvents, combining these polymers with a bilayer membrane introduces complications due to their compatibility. Since PVDF-HFP is a semi-crystalline polymer with low polarity and stronger dispersion forces [26,27] and PSf is an aromatic polymer with strong dipole interactions from the sulfone groups [28], the two polymers have poor miscibility due to their structural differences during bilayer fabrication. The compatibility of the selected polymers with the membrane-forming solvent system was further evaluated using Hansen solubility parameter analysis (Table S1).

Due to their incompatibility, finding an intermediate adhesion material is necessary to improve bonding between these layers. Previous studies have accomplished layer adhesion using poly(methyl methacrylate) (PMMA) and polydopamine (PDA); however, PMMA has fewer desirable properties for membrane applications due to its hydrophobicity [18]. Along with this, employing PMMA as an additive polymer acts as an intense pore former, creating an unnecessary amount of macropores, which can weaken the overall mechanical strength of the membrane [11,29]. Previous studies have shown that PDA enhances layer adhesion but can also increase wrinkling due to its lower crystallinity compared to PMMA. Nevertheless, PDA’s exceptional biocompatibility and biodegradability and lower pore-forming capability make it a valuable option for membrane adhesion applications [17]. The influence of PDA on the thermodynamic stability and phase separation behavior of the membrane-forming systems was further investigated through ternary phase diagram analysis (Figure S2).

#### 2.1.2. Solvent Selection

The selection of solvents in membrane fabrication is important in determining the solubility of the selected polymers, the viscosity of the dope solution, and the overall phase inversion behavior. Eco-friendly solvents, such as Rhodiasolv^®^ PolarClean (PC) and gamma-valerolactone (GVL), have emerged as sustainable alternatives to more traditional organic solvents in membrane fabrication and are used here [21,22,23,24,30,31,32,33,34,35]. PolarClean has been reported as nontoxic and biodegradable, while GVL is bio-based, readily biodegradable, and exhibits low acute toxicity toward aquatic organisms [24,33,34,35]. Accordingly, “eco-friendly” is retained as literature-supported solvent terminology; the present study does not claim that an independent toxicity test or life-cycle assessment was performed. The suitability of the PC/GVL solvent system for the selected polymers was assessed using Hansen solubility parameter analysis and relative energy difference (RED) calculations (Table S1). Furthermore, cloud point measurements were used to evaluate the thermodynamic stability and phase inversion behavior of the polymer–solvent–nonsolvent systems (Figures S1 and S2).

### 2.2. Dope Solution Preparation

Adapting procedures reported by Mulyati et al. [36], polydopamine (PDA) was prepared by mixing 1% dopamine hydrochloride and 0.5% H_2_O_2_ and DI water at room temperature and a stir rate of 400 rpm for 24 h.

The preparation of homogeneous polymer casting solutions, or dope solutions, is an important part of the membrane fabrication process. Complete dissolution of the polymer and additives supports consistent phase inversion behavior and membrane morphology. For this work, the dope solutions were mixed using a stir plate (VWR) at a constant spin rate of 200 rpm for 24 h until a dark-brown solution was obtained. The dark-brown color was treated as a qualitative indication of dopamine oxidation/PDA-containing solution formation, not as spectroscopic confirmation of PDA on the final membrane surface. Dope solutions containing PolarClean and GVL were mixed at 80 °C. Once fully homogenized, dope solutions were cooled to room temperature before analysis and casting. An Elmasonic P water bath sonicator (Elma Schmidbauer GmbH, Singen, Germany) was used to remove residual air bubbles in the solution by sonicating for 15 min. The solutions were stored at room temperature and generally lasted for several months. The original screening used 17 wt% and 22 wt% DBE membranes and 22 wt% SDC bilayer membranes. An additional 17 wt% SDC bilayer was evaluated in the extended-duration study described in Section 2.5.

### 2.3. Casting Process

#### 2.3.1. Single Layer

Single-layer PVDF-HFP and PSf membrane samples were prepared using the doctor blade extrusion (DBE) as described in previous works [22,25,32,37]. In summary, DBE (i.e., blade casting) is a conventionally used method in lab-scale studies to prepare flat sheet membranes involving phase inversion. To prepare membrane sheets using the DBE method, the dope solution was poured onto a glass plate and spread across the plate using a stainless-steel doctor blade (Micrometer Adjustable Film Applicator—250 mm, MTI Corp, Richmond, CA, USA) set with a coating gap of 0.2 mm. In experiments requiring a membrane cast onto a porous support layer, the support layer sheet was taped onto the glass plate, and the solution was cast directly onto the sheet. The casting process then spread the dope solution into a thin film across the plate. The plate was then immersed in a nonsolvent (precipitation) bath containing tap water.

#### 2.3.2. Bilayer

Bilayer casting requires more stability to limit delamination of layers and wrinkling or other defects during the casting process. Unsupported thin-film membranes, especially bilayer membranes, can be fragile and have defects due to the lack of support during casting. To prevent this, along with adding additional mechanical strength and improving the integrity of the membrane, a 4″ × 8″ sheet of nonwoven fiber (polyethylene) support was taped to a glass substrate.

##### Bilayer Using DBE

**Figure 1:**
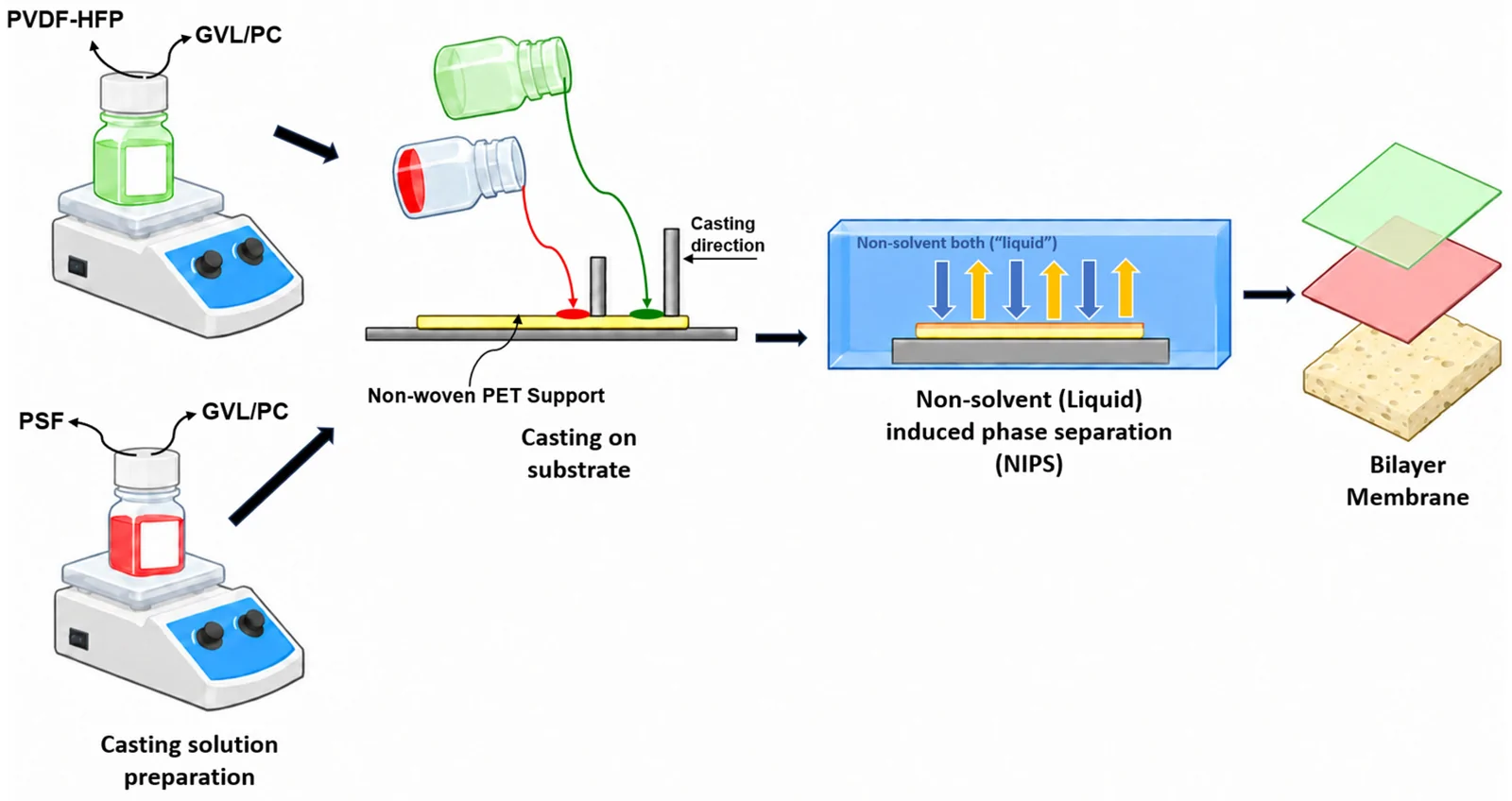
Schematic of the bilayer membrane fabrication process using PVDF-HFP and PSf polymer solutions with adjacent doctor blades.

Each dope solution was first extruded using PSf into a thin film (zero gap) on top of the support via a doctor blade [25]. Once complete, another extrusion, the first PSf layer, was cast on top of the previous thin layer at 20 µm. The cast film and glass substrate were then immersed in a nonsolvent bath of water and allowed to phase invert. After phase inversion occurred, the membranes were stored in a DI water bath for at least 24 h prior to testing. The fabrication process for bilayer membranes was similar, with the only difference being that two doctor blades and two dope solutions were used during extrusion, as shown in Figure 1. Lined up one after the other, the first doctor blade was set to 20 µm, and the blade that follows was set to 25 µm. The larger setting of the two was the top layer of the bilayer membrane, which contained the PVDF-HFP polymer. The smaller setting was the bottom layer of the membrane, which contained the PSf polymer.

Commercial polymeric membranes are commonly prepared on a porous support layer consisting of woven or nonwoven fabric. This support layer provides the membrane with increased mechanical integrity and is generally significantly more porous in structure [38]. This study investigated the wetting of a nonwoven PET fabric support layer with the dope solution. A Hollytex^®^ 3265 nonwoven fabric was selected due to the durability of PET and its reported effects on membrane formation [39].

##### Bilayer Using SDC

The dual-layer slot-die apparatus comprises two slot-die halves along with a central die block. Positioned on each side of the central die block are two PET shims that establish a slot gap of 254 µm for both the bottom and top layers, thus creating two distinct slot gap thicknesses. At the top of the slot-die halves, two inlets facilitate the pumping of membrane dope solutions for each layer. These dope solutions are dispensed concurrently onto a moving substrate, which is composed of glass in this study. This method generates a meniscus that coats the substrate, thereby forming a bilayer coating on its surface. A variety of parameters are meticulously controlled throughout this process, including flow rate, coating gap, substrate speed, and shim thickness. In particular, the flow rate, coating gap, and substrate speed are carefully regulated to ensure that the operating conditions remain within the optimal coating window, thereby minimizing the likelihood of defects during the slot-die operation [20]. The coating gap and substrate speed were kept constant at 290 µm and 5.4 mm/s, respectively. Following the deposition of the bilayer dope solutions, a phase inversion process is employed, similar to that utilized in the DBE process.

### 2.4. Membrane Characterization

One characterization technique used to evaluate the mechanical properties of the fabricated membranes was tensile testing. Young’s modulus measures resistance to tensile deformation and was the mechanical metric available in this study; tensile strength, elongation at break, and toughness were not measured. Using ASTM standard D638-14, membrane samples were cut into dog-bone shapes using a mold to ensure uniform testing conditions. Each strip was then mounted into grips of an Instron 3345 Universal Testing Machine (Illinois Tool Works, Norwood, MA, USA) [40].

Hydrophobicity of a membrane refers to the degree to which a surface resists interaction with water. A goniometer was used to analyze membrane wettability and hydrophilicity by measuring the contact angle a water droplet makes with the membrane surface to characterize the membrane as hydrophobic or hydrophilic. In this study, the goniometer used was the Kruss Drop Shape Analyzer DSA1005 (Matthews, NC, USA).

Membrane morphology was evaluated using scanning electron microscopy (SEM) with a Quanta FEG-250 instrument (Thermo Fisher Scientific, Hillsboro, OR, USA). SEM and SEM-energy-dispersive X-ray spectroscopy were used qualitatively to examine surface morphology, cross-sectional layer continuity, and elemental distribution. Quantitative pore-size distribution, surface porosity, roughness, and charge measurements were not performed.

### 2.5. Filtration Performance

Water flux was calculated as J = V/(A × t), where V is the collected permeate volume, A is the effective membrane area, and t is the filtration time. Experiments used a stainless-steel dead-end cell with an effective membrane area of 13.4 cm^2^ at 414 kPa (60 psi; 4.14 bar). Water permeability was calculated as Lp = J/ΔP and is reported in LMH/bar. Solute rejection was calculated as R = (1 − Cp/Cf) × 100%, where Cp and Cf are the permeate and feed concentrations. Table 2 shows the tested solutes and initial feed concentrations. To begin addressing extended-duration operation, additional tests were conducted using representative 17 wt% DBE and 17 wt% SDC bilayer membranes. Sequential pure-water measurements used 5 mL collections, and 100 ppm BSA filtration used 4 mL samples collected at 0, 1, 4, 8, 16, and 24 h. Complete data and plots are provided in Supplementary Section S3.

## 3. Results

### 3.1. Scanning Electron Microscopy (SEM)

**Figure 2:**
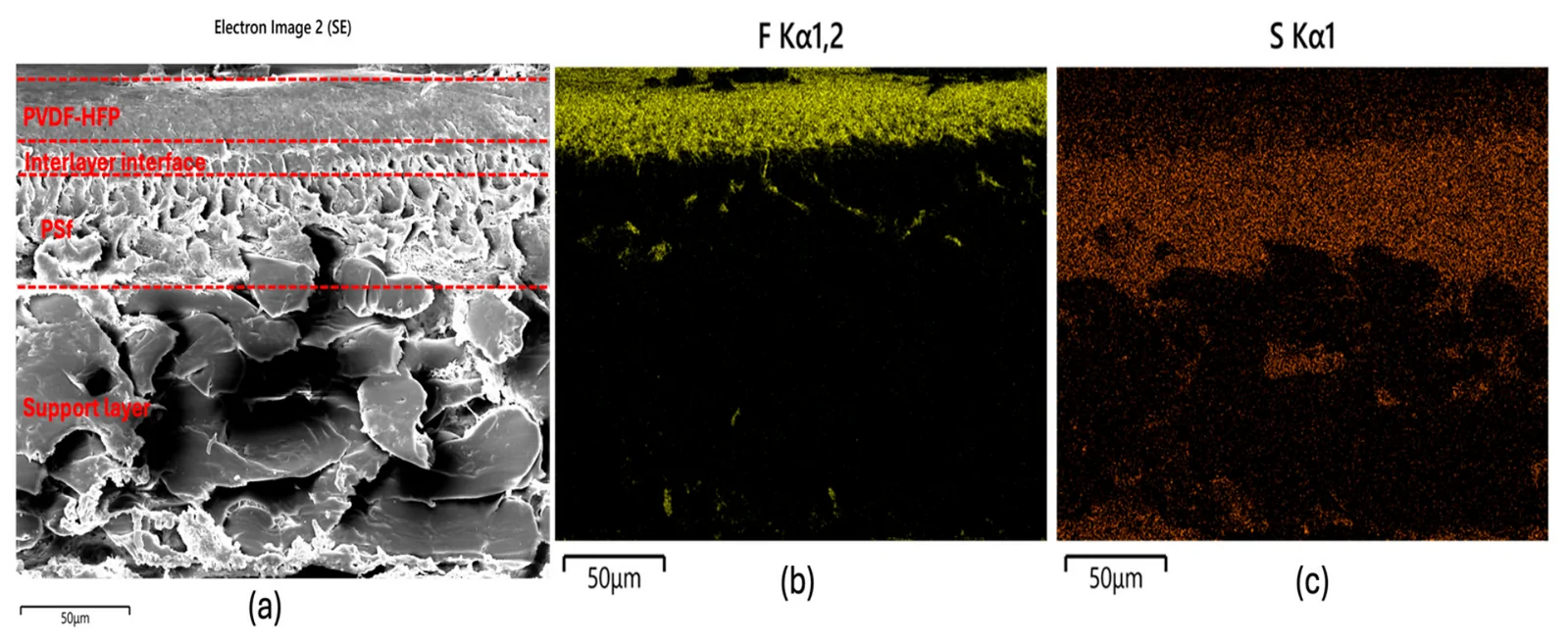
(**a**) SEM image of the bilayer membrane cross section; (**b**) F elemental mapping to show the PVDF top layer; and (**c**) S elemental mapping to show the PSf bottom layer.

Cross-sectional SEM images were obtained to identify the PVDF-HFP top layer, PSf bottom layer, and support. SEM combined with energy-dispersive X-ray spectroscopy (SEM-EDS) mapped fluorine as an identifiable element for PVDF-HFP and sulfur for PSf. The PVDF-HFP and PSf layers were in continuous contact without a visible interfacial gap in the imaged section, providing qualitative evidence of layer continuity but not a quantitative measurement of adhesion strength. As shown in Figure 2, fluorine was concentrated in the upper layer, while sulfur was concentrated below it. Small streaks of fluorine in the PSf region may reflect PVDF-HFP penetration into pores, and sulfur was also observed within voids of the support. The elemental maps support the assignment of the PVDF-HFP and PSf regions and qualitative interfacial continuity.

No visible interfacial gap was observed in the cross-sectional SEM images (Figure 2), which is consistent with layer continuity in the imaged region. This observation agrees with the Hansen solubility analysis (Table S1), where the PVDF-HFP/PDA/PC/GVL and PSf/PDA/PC/GVL systems exhibited RED values of 0.287 and 0.688, respectively. RED values below 1 indicate favorable polymer–solvent compatibility during membrane formation; however, no-PDA controls and peel-strength or delamination tests were not performed, so the images do not establish quantitative adhesion strength.

**Figure 3:**
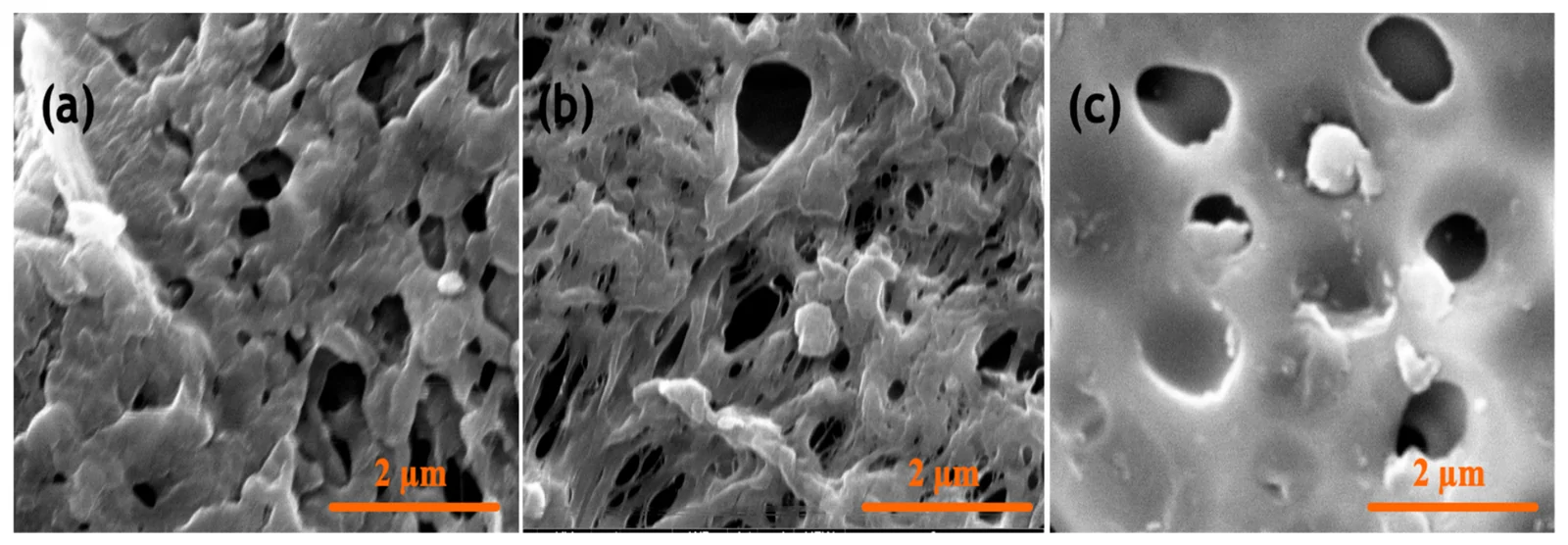
Surface scanning electron microscopy (SEM) images of single-layer (**a**) PSf, (**b**) PVDF-HFP, and (**c**) bilayer membranes at a magnification of 2 µm.

In addition, SEM was performed on the surface of the membranes to determine their pore structure and uniformity. This information helps explain membrane morphology and can be used to explain trends in membrane performance, such as flux and ion rejection. The surface images of 17 wt% single-layer PSf and PVDF-HFP supported membranes, along with the supported bilayer membrane, are shown in Figure 3.

Due to the hexafluoropropylene (HFP) comonomer in PVDF-HFP, the crystallinity within PVDF is reduced, which reduces the uniformity of the PVDF structure (Figure 3b). PVDF on its own is semicrystalline, with both crystalline and amorphous regions [32]. Crystallization in a polymer allows for pore walls to be interconnected within the membrane, and the pores themselves become elongated and less uniform with the addition of HFP. HFP causes a slight delay in the rate of demixing (when compared to PSf), which also contributes to the elongated, less uniform pore structure. Along with this, HFP increases the polymer to become more polar [32].

The surface SEM imagery (Figure 3) results can also be explained when comparing the thermodynamic properties of both PVDF-HFP and PSf membranes. PSf is a completely amorphous polymer, meaning it possesses no crystalline properties [28]. The lack of crystallinity yields a slightly higher rate of demixing (when compared to PVDF-HFP), so the pores are tighter and not elongated like PVDF-HFP membranes. PSf has a slightly lower polymer–solvent affinity when compared to PVDF-HFP, and its polymer–nonsolvent affinity is very low due to the sulfone group found in PSf being polar. When viewing a ternary phase diagram for PSf or a similar substance in polyethersulfone (PES), the binodal curve is closer to the polymer–solvent axis, and the spinodal curve is close to the binodal curve [41]. This yields a smaller metastable region, which means there is a higher rate of demixing. This higher rate of demixing causes the phase inversion time during NIPS to be quicker, leading to a more uniform pore structure.

PVDF-HFP, on the other hand, possesses higher polymer–solvent affinity along with a low polymer–nonsolvent affinity. When viewing a ternary phase diagram, the binodal curve is closer to the nonsolvent axis, which indicates a slight delay before the phase separation occurs during NIPS. The spinodal curve is pushed farther inward on the diagram, leading to a larger metastable region. When the metastable region is larger, there is a delayed rate of demixing. The phase inversion time with PVDF-HFP membranes is slower [42] than PSf membranes because of this, leading to an elongated pore structure [43]. The bilayer membrane surface morphology (Figure 3c) is similar to that of the PVDF-HFP single-layer membrane due to the PVDF-HFP layer being the top layer of the bilayer. Greater apparent pore blockage was observed in the bilayer membrane, along with bright features within the pore structure. However, the chemical identity of these features was not independently confirmed, and therefore they cannot be definitively assigned to PDA based on SEM imaging alone.

### 3.2. Young’s Modulus

Mechanical testing, using Young’s modulus, was completed to investigate the effects of the support and polymer selection. A higher Young’s modulus indicates greater resistance to tensile deformation, while a lower value indicates greater flexibility; however, modulus alone does not establish overall durability or resistance to cracking [44]. The membranes were cast on the nonwoven support [25]. A comparison of Young’s modulus across polymer types and between single- and bilayer eco-friendly membranes is shown in Table 3.

Table 3 depicts the results of the Young’s modulus tests completed on supported eco-friendly single- and bilayer membranes. Polysulfone (PSf) single-layer membranes yielded a higher Young’s modulus than PVDF-HFP single-layer membranes, indicating greater resistance to tensile deformation under the tested conditions. This may reflect the aromatic structure and strong intermolecular interactions of PSf. PVDF-HFP has lower crystallinity than PVDF because HFP comonomers disrupt ordered packing [45], which may contribute to greater flexibility [46]. The bilayer membrane exhibited the highest Young’s modulus among the tested samples; however, Young’s modulus alone does not establish complete mechanical durability.

### 3.3. Hydrophobicity

**Figure 4:**
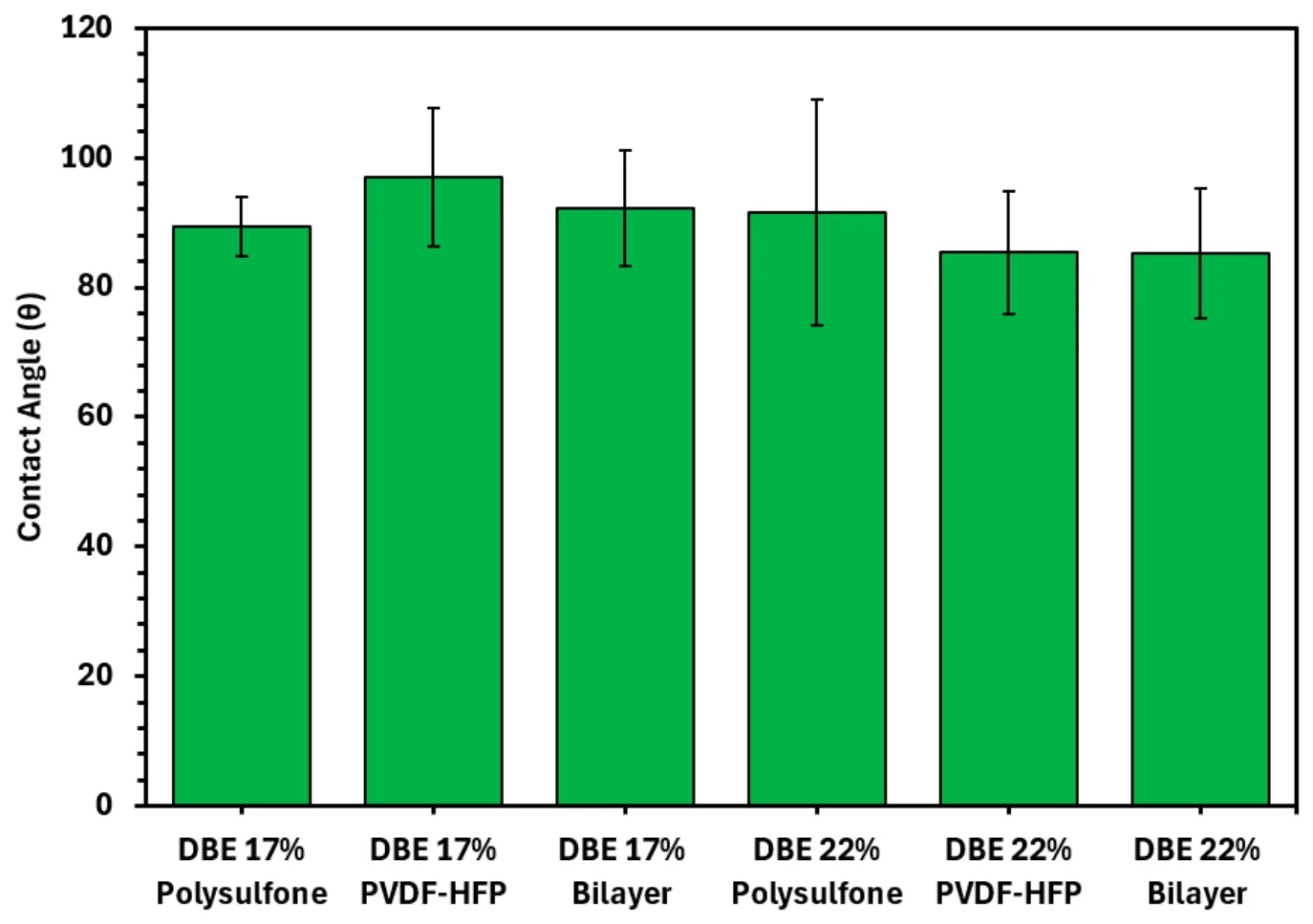
Hydrophobicity of 17 wt% and 22 wt% PSf, PVDF-HFP and bilayer membranes.

Water contact angles, shown in Figure 4, were similar across the single- and bilayer membranes. Because an inferential statistical test was not reported, the differences are interpreted as descriptive trends rather than statistically distinct groups. PVDF-HFP contains fluorinated C–F bonds and PSf contains aromatic and sulfone groups, both of which influence surface wetting. PDA may be distributed within the dope or pores rather than forming a continuous top-surface coating, which could limit its effect on the measured contact angle. Polymer concentration produced only small contact-angle differences within the observed variability.

### 3.4. Membrane Permselectivity

#### 3.4.1. Water Permeability

**Figure 5:**
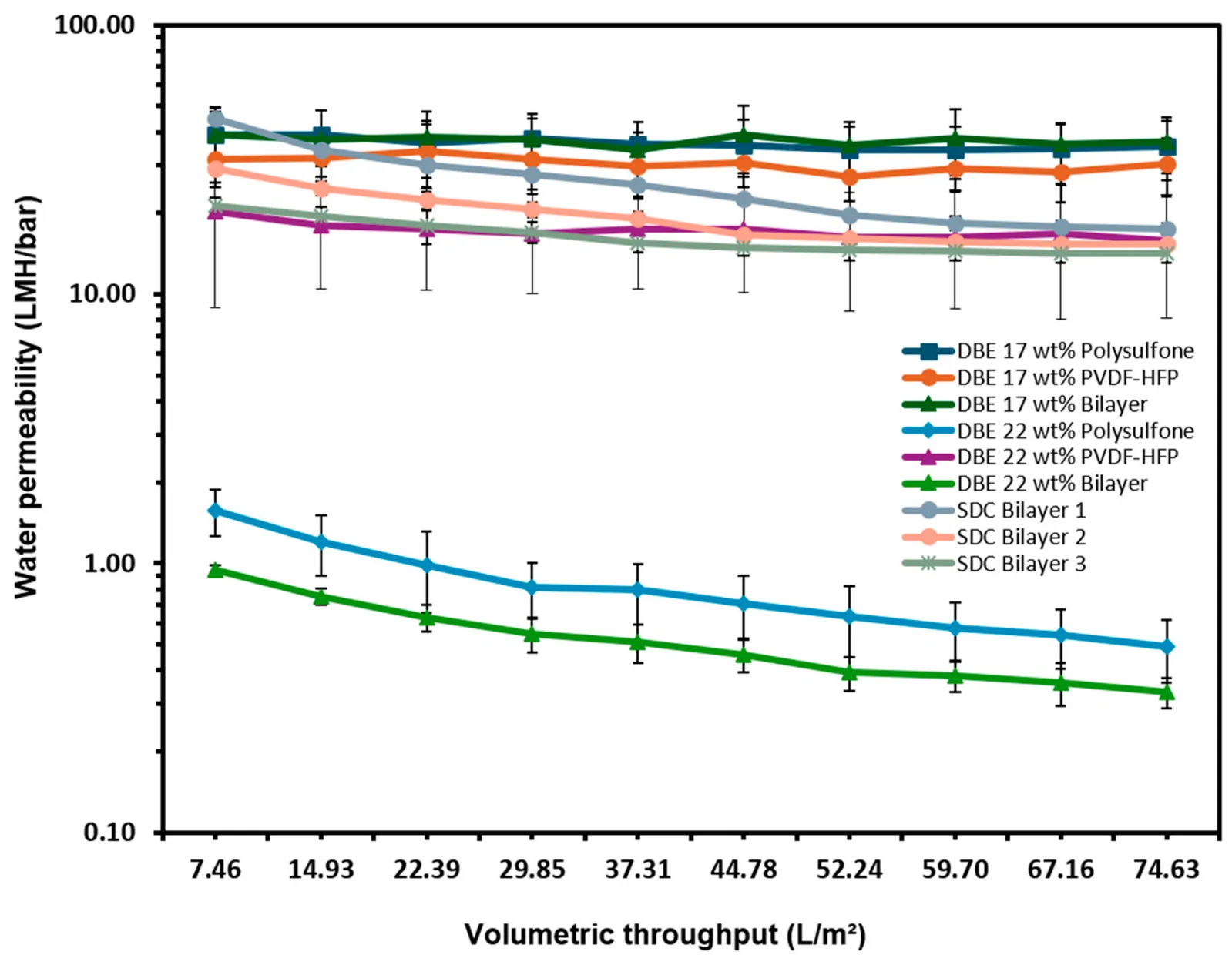
Water permeability of DBE and SDC membranes as a function of volumetric throughput at 60 psi (4.14 bar). The y-axis is logarithmic. Points represent means of three membrane replicates; error bars indicate sample standard deviations (*n* = 3).

To assess membrane performance, filtration experiments were performed on the nine membrane types fabricated here. Three membranes consisted of 17 wt% polymer and were fabricated using DBE, PSf, PVDF-HFP, and a bilayer, each evaluated in triplicate. The bilayer contained a PVDF-HFP top layer and a PSf bottom layer. A second set contained the corresponding 22 wt% DBE membranes. Finally, three SDC bilayer membranes were fabricated using PVDF-HFP top-layer flow rates of 0.86, 0.72, and 0.61 mL/min and are referred to as SDC Bilayers 1, 2, and 3, respectively. Pressure-normalized water permeability values at 60 psi (4.14 bar) are shown in Figure 5 as a function of volumetric throughput on a logarithmic y-axis.

The DBE 17 wt% membranes showed the highest and most stable water permeability, generally approximately 27–39 LMH/bar. The SDC bilayers began at approximately 21–45 LMH/bar and declined to approximately 14–18 LMH/bar across the volumetric-throughput range of 7.46–74.63 L/m^2^. The DBE 22 wt% PVDF-HFP membrane remained near 16–20 LMH/bar, whereas the DBE 22 wt% PSf and bilayer membranes showed the lowest values, approximately 0.5–1.6 LMH/bar. Thus, the data support a polymer-concentration effect but do not support describing all SDC membranes as the lowest-permeability group.

Lower polymer concentration increases the solvent fraction and generally reduces dope viscosity. During NIPS, this can accelerate the solvent–nonsolvent exchange and promote macrovoid formation and heterogeneous pore structures [47,48,49,50]. Such morphology may contribute to the higher permeability and variability observed for some 17 wt% DBE membranes, although demixing kinetics and pore-size distributions were not measured directly. Higher-viscosity solutions can slow exchange and may produce more controlled structures; this interpretation should be confirmed by quantitative porometry or image analysis.

Differences in permeability variability among PSf and PVDF-HFP membranes may reflect their distinct polymer–solvent and polymer–nonsolvent interactions. PSf interacts strongly with polar solvents such as PolarClean and GVL [31], whereas the semicrystalline PVDF-HFP system can exhibit delayed demixing and elongated pore structures [27,47]. These relationships are consistent with the qualitative SEM observations but do not directly prove the transport mechanism.

The DBE 22 wt% PSf and bilayer membranes displayed the lowest water permeability values. The two-layer structure may influence phase inversion and transport resistance, but the present SEM images do not quantify pore-size distribution or transport-pathway uniformity.

The SDC bilayers occupied an intermediate permeability range and decreased with increasing throughput. Slot-die coating provides controlled metering of flow rate, coating gap, and substrate speed [20], but the present results alone do not establish that SDC always produces lower variability than DBE. Compaction, fouling, and layer morphology may contribute to the observed decline and require dedicated testing.

#### 3.4.2. Solute Rejection

**Figure 6:**
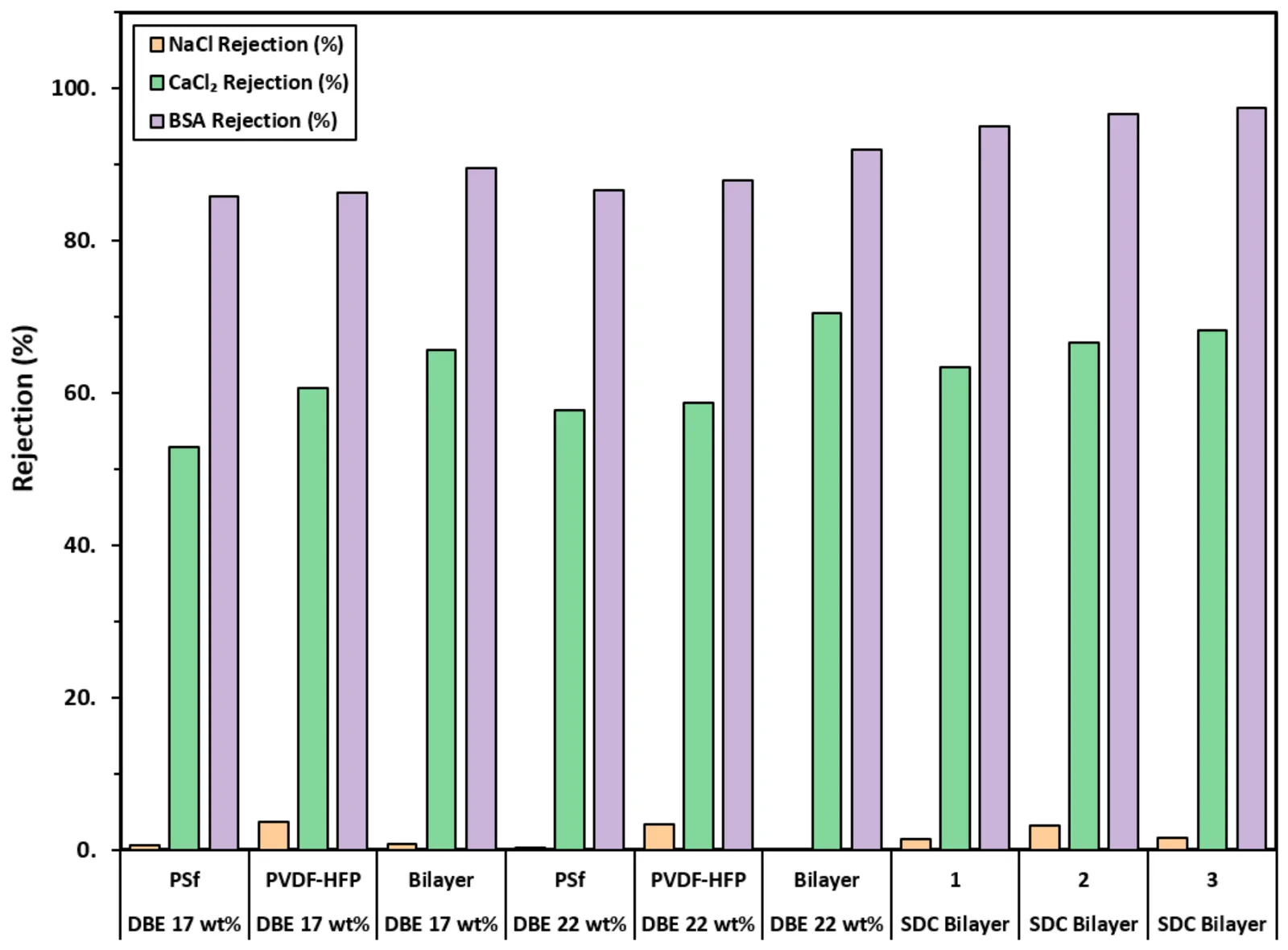
Solute rejection of DBE and SDC membranes.

To evaluate membrane performance and compare the pore formation between membranes, ion rejection experiments were completed across supported single and bilayer membranes. Initially, sodium chloride was selected as the feed solution to simulate reverse osmosis behavior to determine if the membranes tested here were able to achieve monovalent ion rejection. Sodium chloride possesses monovalent sodium ions that have hydrodynamic radii of 3.6 Angstroms (~0.36 nm) and a hydration number of 4–6 water molecules [51]. Sodium chloride solutions at 100 ppm were filtered through each membrane type, and a conductivity meter was used to assess sodium ion rejection. All rejection values for all single and bilayer membranes with both SDC and DBE methods were below 10%, with most nearing no rejection. This shows that the pore sizes of the membranes were larger than the hydrodynamic radius of sodium molecules, and thus even the bilayer membranes could not emulate reverse osmosis behavior. Filtrations were followed with divalent ions and BSA protein. Results are shown in Figure 6.

Since monovalent ions were not filtered by the membranes, similar experiments were performed using divalent ions in the form of calcium chloride to determine if the bilayer membranes could simulate nanofiltration behavior. Divalent ions have hydrodynamic radii of 4.1 Angstroms (~0.41 nm) and a hydration number of 6–8 water molecules [52]. Divalent cations in the form of CaCl_2_ at 100 ppm were filtered through the membranes, and the results are shown in Figure 6. The bilayer DBE membranes (17 wt% and 22 wt%) displayed rejections of approximately 65% and over 70%, respectively, compared with their single-layer counterparts. The SDC bilayers rejected approximately 63–68%, and rejection increased as the PVDF-HFP dope flow rate decreased. These trends are consistent with changes in effective transport pathways, but because the zeta potential was not measured, the data cannot distinguish size-exclusion effects from possible charge/Donnan contributions.

To evaluate membrane performance and compare pore formation between membranes fabricated with varying polymer concentrations and methods, rejection experiments using bovine serum albumin (BSA) were performed. BSA is a globular protein derived from cow blood that is commonly used as a model macromolecule in membrane filtration studies. It is larger than monovalent and divalent ions, measured to be approximately 69 kilodaltons [53]. The BSA rejection results of the same nine membranes fabricated using DBE and SDC methods are shown in Figure 6.

The SDC bilayer membranes exhibited the highest initial BSA rejection (Figure 6). SDC Bilayer 3 reached approximately 97%, followed by SDC Bilayers 2 and 1. Within the SDC group, Bilayer 3 combined the lowest water permeability with the highest BSA rejection. The DBE 17 wt% single-layer PSf and PVDF-HFP membranes showed approximately 85–86% rejection, while the DBE 17 wt% bilayer reached almost 89%. The DBE 22 wt% bilayer reached approximately 92% and exceeded its single-layer counterparts. These results are consistent with greater transport resistance in the bilayer structures, although quantitative pore-size and surface-charge data would be required to establish the separation mechanism. The more controlled SDC process may contribute to consistent rejection, but the present data do not isolate casting methods from formulation and layer-structure effects.

To begin addressing operational stability, additional filtration was performed with representative 17 wt% DBE and 17 wt% SDC bilayer membranes. During sequential pure-water testing over cumulative throughput from 3.73 to 44.78 L/m^2^, DBE permeability changed from 10.43 to 8.40 LMH/bar and SDC permeability from 8.74 to 5.73 LMH/bar. During 24 h filtration of 100 ppm BSA, DBE rejection increased from 81.58% to 89.58% and permeability decreased from 6.59 to 0.80 LMH/bar; SDC rejection increased from 86.39% to 95.06% and permeability decreased from 5.87 to 0.74 LMH/bar. These single-membrane profiles provide an initial extended-duration assessment but do not establish full long-term durability. Complete measurements and plots are provided in Supplementary Section S3.

## 4. Conclusions

The primary objective of this work was to investigate the fabrication and performance of eco-friendly polymeric membranes for water treatment applications, with a focus on eco-friendly bilayer membranes. Single-layer and bilayer membranes were successfully fabricated using PolarClean/GVL, a solvent system described as green in the cited literature. A comparative analysis of two NIPS casting techniques, DBE and SDC, highlighted differences in membrane fabrication and performance. Polydopamine was used as an adhesion-promoting formulation component, while SEM-EDS provided qualitative evidence of layer continuity rather than quantitative adhesion strength. Performance testing showed divalent-ion rejection of 52–70% and BSA rejection of 85–97% across the initial membrane screening. In an additional 24 h BSA filtration, rejection increased from 81.58% to 89.58% for the representative 17 wt% DBE bilayer and from 86.39% to 95.06% for the representative 17 wt% SDC bilayer, while permeability decreased from 6.59 to 0.80 and from 5.87 to 0.74 LMH/bar, respectively. These data provide an initial extended-duration assessment but do not replace longer cycling, fouling-recovery, or adhesion-durability studies.

## Figures and Tables

**Table 1:** Literature context for eco-friendly solvent membrane fabrication, bilayer membrane design, and scalable coating relative to the present study.

Prior Focus	System/Route	Feed or Application	Metrics Emphasized	Relevance to Present Study
PVDF-HFP membranes from PolarClean	PVDF-HFP/PolarClean; VIPS/NIPS with PolarClean	Water-treatment-oriented membrane testing	Morphology, porosity, wettability, pore size, roughness, and mechanical properties	Supports the feasibility of PolarClean for PVDF-HFP but does not address PVDF-HFP/PSf PDA-containing bilayers or SDC translation [21].
PSf membranes from PolarClean/GVL	PSf/PolarClean/GVL; NIPS with low-hazard solvent/cosolvent systems	Aqueous filtration	Flux, rejection, and morphology	Supports the use of PolarClean/GVL with PSf and motivates mixed-solvent selection [22,23].
GVL as bio-based NF solvent	CA, CTA, PI, PES, PSU in GVL; NIPS using water or ethanol nonsolvent	Rose Bengal and MgSO_4_ model feeds	Permeability, rejection, viscosity, cloud point, and morphology	Shows that GVL-based casting can be rationalized through HSP, viscosity, and cloud-point analysis [24].
Bilayer PVDF/PVDF-PSF membranes	PVDF/PVDF-PSF; Bilayer fiber-aligned membranes using green solvent	Membrane distillation	Hydrophobicity and distillation performance	Demonstrates green-solvent bilayer PVDF/PSF concepts, but in a different application and structure [10].
Dual-layer membrane fabrication review	Organic dual-layer hollow fibers; Co-extrusion and phase inversion	Water treatment and gas separation	Mechanistic and processing challenges	Frames the broader bilayer membrane challenge of interfacial compatibility and scalable fabrication [16].
R2R/SDC membrane scale-up	PSf/PolarClean/GVL supported membranes; Slot-die/R2R coating	Loose nanofiltration	Coating window, support wetting, flux, and selectivity	Motivates SDC as a scalable route but does not examine PDA-containing PVDF-HFP/PSf bilayers [25].

**Table 2:** Solutes tested for rejection experiments and initial feed concentrations.

Solute Tested	Type of Contaminant	Feed Concentration	Size of Solute
Sodium Chloride (NaCl)	Monovalent Ion	100 ppm	0.36 nm
Calcium Chloride (CaCl_2_)	Divalent Ion	100 ppm	0.41 nm
Bovine Serum Albumin (BSA)	Protein	100 ppm	7.1 nm

**Table 3:** Average Young’s modulus (in MPa) of membranes with varying polymer types, with standard deviation.

	PSf/PolarClean/GVL	PVDF-HFP/PolarClean/GVL	Bilayer PolarClean/GVL
Young’s Modulus (MPa)	196.6 ± 3.93	159.6 ± 2.01	216.3 ± 11.53

## Data Availability

The raw data supporting the conclusions of this article will be made available by the authors on request.

## Supplementary Materials

The following supporting information can be downloaded at: https://www.mdpi.com/article/10.3390/membranes16090300/s1, Figure S1, Ternary phase diagram of a PVDF/NMP/water and PVDF-HFP/NMP/water system; Figure S2, Ternary phase diagram of (a) PVDF-HFP/PDA/PC/GVL/water and (b) PSf/PDA/PC/GVL/water systems; Figure S3, Extended-duration filtration performance of representative 17 wt% bilayer membranes fabricated by doctor-blade extrusion (DBE) and slot-die coating (SDC) at 414 kPa (60 psi): (a) pure-water permeability as a function of volumetric throughput, (b) permeability during 100 ppm BSA filtration, and (c) BSA rejection during the 24 h filtration period; Table S1, Hansen solubility parameters and compatibility analysis of the membrane-forming systems; Supplementary methods and analyses draw on previously published procedures and supporting literature [21,23,54,55,56,57,58,59,60].
