# Supplementary material for: Eco-Friendly Solvent-Based Fabrication of Single-Layer and Polydopamine-Modified Bilayer PVDF-HFP Membranes
Source: Membranes (Basel). 2026 Sep 13;16(9):300. doi: 10.3390/membranes16090300 (PMC13609762; doi:10.3390/membranes16090300)
Supplement: Supplementary file 1 [file membranes-16-00300-s001.zip › membranes-4471541-supplementary.pdf]

## Supplementary Materials

# Eco-Friendly Solvent-Based Fabrication of Single-Layer and Polydopamine-Modified Bilayer PVDF-HFP Membranes

**Tridwip Sen [1,†], Eesh Kulshrestha [1,†], Muhammad Usman Yousaf [1], Minwoo Jung [2], Tequila A. L. Harris [2,*] and Isabel C. Escobar [1,*]**

[1] Department of Chemical and Materials Engineering, Stanley and Karen Pigman College of Engineering, University of Kentucky, Lexington, KY 40506, USA
[2] George W. Woodruff School of Mechanical Engineering, Georgia Institute of Technology, Atlanta, GA 30332, USA
* Correspondence: authors: tharris3@gatech.edu (T.A.L.H.); isabel.escobar@uky.edu (I.C.E.)
† These authors contributed equally to this work.

## S1. Thermodynamic Study

*S1.1. Hansen Solubility Study*

Using the Hansen solubility sphere theory, the relative affinity between a polymer and solvent, $R_a$, was calculated as follows:

$$R_a = \sqrt{4(\delta_{d2} - \delta_{d1})^2 + (\delta_{p2} - \delta_{p1})^2 + (\delta_{\mathrm{h}2} - \delta_{\mathrm{h}1})^2} \quad (S1)$$

where $\delta_d$, $\delta_p$, and $\delta_{\mathrm{h}}$ represent the dispersion, polar, and hydrogen bonding parameters, respectively; 1 and 2 represent the polymer and solvent components. The relative energy difference, $RED$, between polymer and solvent is found using equation 12 below, where $R_o$ represents the interaction radius of the polymer sphere[23].

$$RED = \frac{R_a}{R_o} \quad (S2)$$

In a cosolvent system, the respective contribution from each solvent towards the dispersion, polar, and hydrogen bonding force parameters can be calculated using the volume fractions, $V_i$, of the solvent mixture, as shown below in equations 3-6. Volume fractions were calculated for each species using equation 13, where $W_i$ represents weight fraction and $\rho_i$ represents density of the respective species[54].

$$V_i = \frac{\frac{W_i}{\rho_i}}{\frac{W_1}{\rho_1} + \frac{W_2}{\rho_2}} \quad (S3)$$

$$\delta_d = V_1\delta_{d1} + V_2\delta_{d2} \quad (S4)$$

$$\delta_p = V_1\delta_{p1} + V_2\delta_{p2} \quad (S5)$$

$$\delta_h = V_1\delta_{h1} + V_2\delta_{h2} \quad (S6)$$

The relative energy difference (RED) is used to evaluate polymer–solvent compatibility, where RED < 1 indicates good solubility and favorable interactions, while RED > 1 suggests poor compatibility and a greater tendency for phase separation. Lower RED values correspond to stronger polymer–solvent affinity and increased thermodynamic stability [23]. The calculated solubility parameters, Hansen distances ($R_a$), and RED values

for the investigated systems are summarized in Table S1. Both membrane-forming systems exhibited RED values below 1, confirming favorable compatibility with the PC/GVL solvent mixture. The lower RED value of the PVDF-HFP/PDA/PC/GVL system (0.287) compared to the PSf/PDA/PC/GVL system (0.688) indicates stronger polymer–solvent interactions and greater thermodynamic stability.

**Table S1.** Hansen solubility parameters and compatibility analysis of the membrane-forming systems.

| Material | Type | δP | δH | δP | $R_0$ | Polymer/ Solvent System | Ra | RED |
|---|---|---|---|---|---|---|---|---|
| **PSf [55]** | Polymer | 18.5 | 8.2 | 7.1 | 8.0 | PSf/PDA/ PC/GVL | 6.07 | 0.688 |
| **PVDF-HFP [55]** | Polymer | 17.2 | 12.5 | 9.2 | 8.1 | | | |
| **Polar Clean (PC) [56]** | Solvent | 15.8 | 10.7 | 9.2 | | | | |
| **Gamma-valerolactone (GVL) [56]** | Solvent | 16.7 | 14.0 | 8.0 | | PVDF-HFP/ PDA/PC/GV | 2.79 | 0.287 |
| **Polydopamine (PDA) [57]** | Polymer (Adhesive) | 18.2 | 9.0 | 17.1 | 26.55 | | | |
| **Water [57]** | Nonsolvent | 15.5 | 16.0 | 42.3 | | | | |

## S2. Cloud Point Curve

Cloud point curve determination was conducted using the titration method [58]. Solutions of varying polymer weight percent were prepared using NMP as the solvent and allowed to stir for 24h for full polymer dissolution. PVDF solutions included 8, 10, 12, 14, and 16 wt.% in NMP and PVDF-HFP solutions included 10,12, 14, 16, and 18wt.% in NMP [21]. These solutions were then titrated with deionized water while being continuously stirred at room temperature until turbidity was observed. The solutions were then allowed to stir for an additional 24h to ensure cloud point had been reached. The known values of polymer weight, solvent volume, and microliters of water added were then converted to volume percentages. The ternary phase diagram was plotted using OriginPro.

To better understand the thermodynamics, cloud point diagrams were constructed using PVDF and PVDF-HFP copolymer dissolved in NMP, titrated with water as the nonsolvent. The ternary phase diagram is shown below in Figure S1. The PVDF cloud point curve was conducted in triplicate and corresponded with curves found in literature [59]. The relative linearity of this curve indicates that the amount of water required to induce phase separation increases linearly with polymer concentration, as expected [60]. This is likely due to solution viscosity increasing because of increased polymer concentration, and therefore, slower diffusion rates. The cloud point curve of the co-polymer system is shifted towards the polymer/nonsolvent axis; that is, the composition path increased, indicating that a greater amount of water is required to destabilize the solution and induce demixing. This shift shows the co-polymer produces a more thermodynamically stable solution under the set conditions.

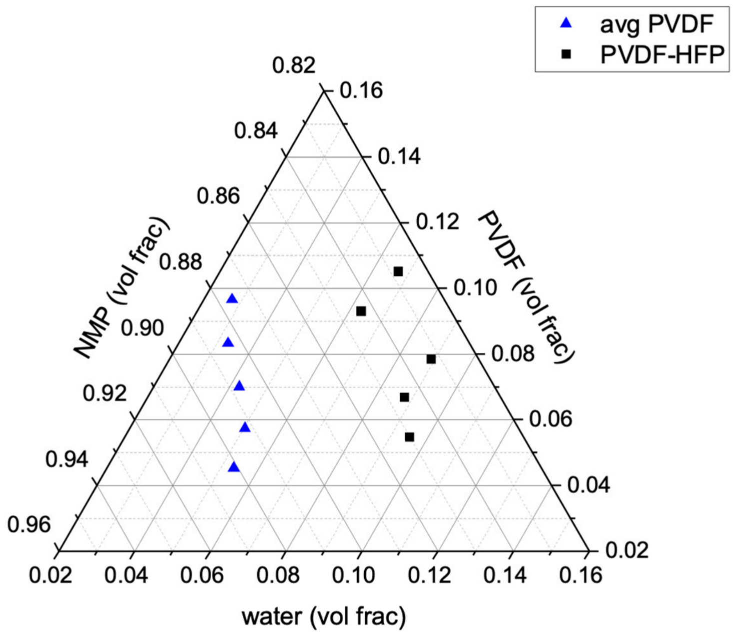


**Figure S1.** Ternary phase diagram of a PVDF/NMP/water and PVDF-HFP/NMP/water system.

Further insight into the phase inversion behavior of the membrane-forming systems was obtained from the ternary phase diagrams shown in Figure S2. The cloud point compositions for both the PVDF-HFP/PDA/PC/GVL/water and PSf/PDA/PC/GVL/water systems define the onset of liquid-liquid demixing and the boundary between the stable and metastable regions. Compared with the PVDF-HFP system discussed previously, the incorporation of polydopamine (PDA) altered the phase separation behavior of the casting solutions. The PVDF-HFP/PDA system exhibited cloud points at lower water fractions, indicating that the solution reached the demixing boundary with a relatively small amount of nonsolvent addition. In contrast, the PSf/PDA system showed a shift toward higher water fractions, demonstrating greater resistance to phase separation and therefore increased thermodynamic stability under the investigated conditions. This behavior suggests stronger interactions between PSf, PDA, and the PC/GVL solvent mixture, which delayed the onset of demixing. The differences in cloud point composition are expected to influence membrane formation kinetics and ultimately contribute to variations in pore structure and morphology.

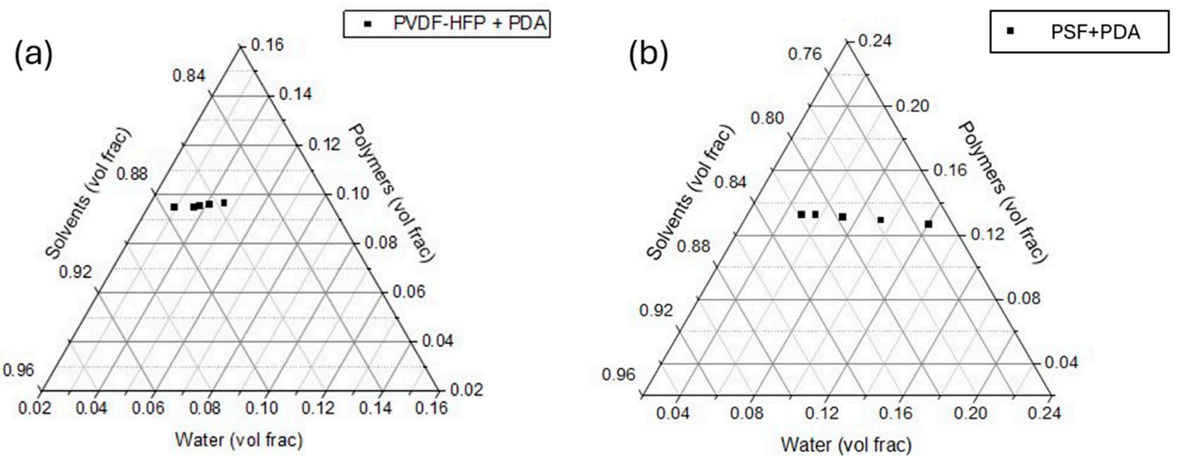


**Figure S2.** Ternary phase diagram of (a) PVDF-HFP/PDA/PC/GVL/water and (b) PSF/PDA/PC/GVL/water system.

## S3. Extended-Duration Filtration Performance

To provide an initial assessment of performance during extended operation, representative 17 wt% DBE and 17 wt% SDC bilayer membranes were tested at 414 kPa (60 psi) in a stainless-steel dead-end cell with an effective membrane area of 13.4 cm². Pure-water permeability was monitored with increasing volumetric throughput, and 100 ppm BSA filtration was evaluated for 24 h. Each point represents one membrane measurement (n=1); thus, the results are interpreted as preliminary extended-duration profiles rather than definitive evidence of long-term durability.

Across a cumulative throughput of 3.73–44.78 L/m² (LMH), DBE pure water permeability decreased from 10.43 to 8.40 LMH/bar, while SDC pure water permeability decreased from 8.74 to 5.73 LMH/bar, as shown in Figure S3a. DBE maintained the higher permeability throughout the test, and the magnitude of change became smaller during the later collections for both membranes. During 24 h filtration of 100 ppm BSA, as shown in Figure S3b, the permeability decreased from 6.59 to 0.80 LMH/bar for DBE and from 5.87 to 0.74 LMH/bar for SDC. The largest decline occurred during the early filtration period, and the values converged by 24 h. This behavior is consistent with increasing hydraulic resistance during protein filtration, although the present measurements do not distinguish among membrane compaction, concentration polarization, and BSA deposition. Lastly, BSA rejection increased from 81.58% to 89.58% for DBE and from 86.39% to 95.06% for SDC (Figure S3c). The SDC membrane maintained higher rejection at every sampling time. The simultaneous increase in rejection and decrease in permeability demonstrates improved protein retention accompanied by a substantial loss of hydraulic transport during the 24 h test.

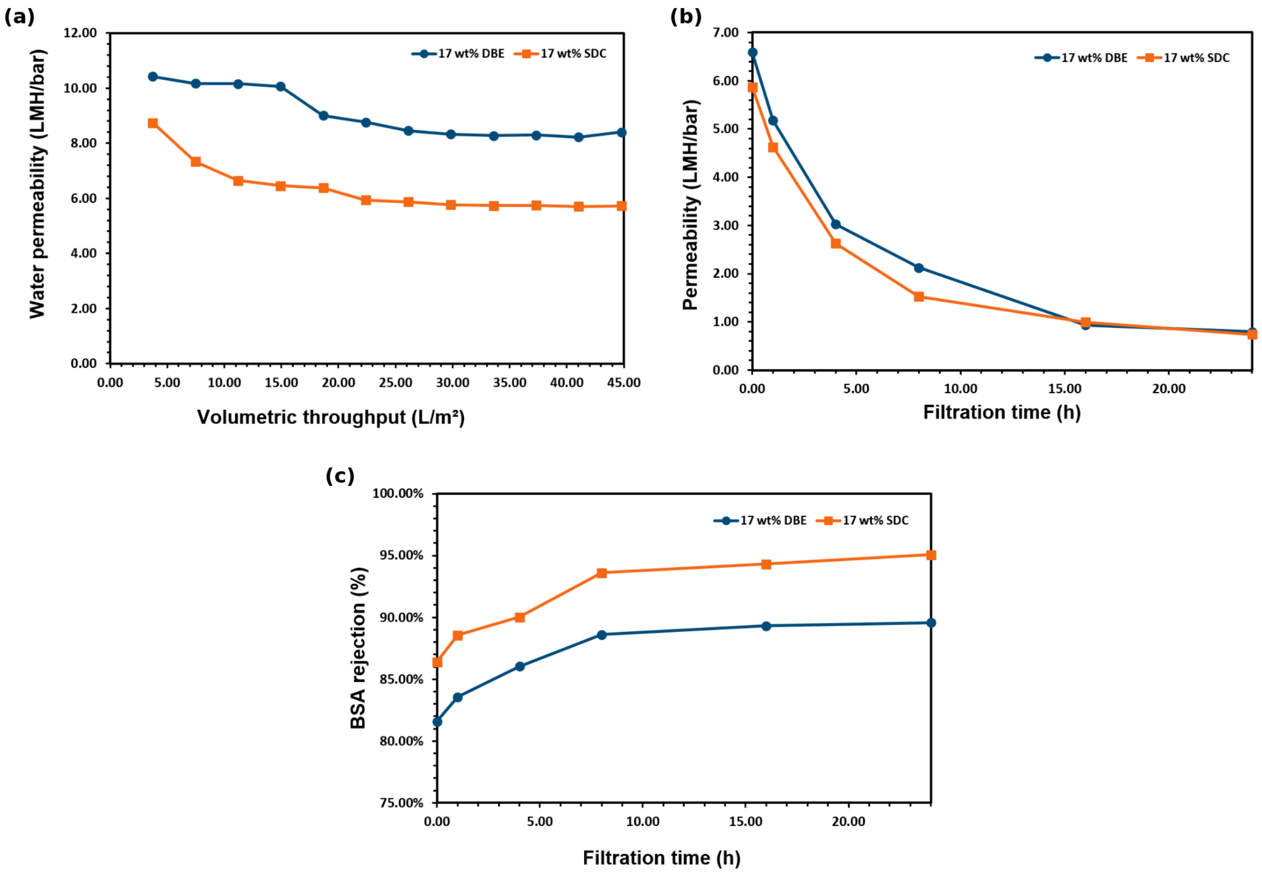


**Figure S3.** Extended-duration filtration performance of representative 17 wt% bilayer membranes fabricated by doctor blade extrusion (DBE) and slot-die coating (SDC) at 414 kPa (60 psi): (a) pure-

water permeability as a function of volumetric throughput; (b) permeability during 100 ppm BSA filtration; and (c) BSA rejection during the 24 h filtration period.

## References:


21. Russo, F.; Ursino, C.; Sayinli, B.; Koyuncu, I.; Galiano, F.; Figoli, A. Advancements in Sustainable PVDF Copolymer Membrane Preparation Using Rhodiasolv® PolarClean as an Alternative Eco-Friendly Solvent. *Clean Technol.* **2021**, *3*, 761–786. https://doi.org/10.3390/cleantechnol3040045.

23. Dong, X.; Shannon, H.D.; Parker, C.; De Jesus, S.L.; Escobar, I.C. Comparison of Two Low-Hazard Organic Solvents as Individual and Cosolvents for the Fabrication of Polysulfone Membranes. *AIChE J.* **2020**, *66*, e16790. https://doi.org/10.1002/aic.16790.

54. Xie, W.; Li, T.; Chen, C.; Wu, H.; Liang, S.; Chang, H.; Liu, B.; Drioli, E.; Wang, Q.; Crittenden, J.C. Using the Green Solvent Dimethyl Sulfoxide to Replace Traditional Solvents Partly and Fabricating PVC/PVC-g-PEGMA Blended Ultrafiltration Membranes with High Permeability and Rejection. *Ind. Eng. Chem. Res.* **2019**, *58*, 6413–6423.

55. Zhu, H. Interfacial Preparation of Ferroelectric Polymer Nanostructures for Electronic Applications. *Polym. J.* **2021**, *53*, 877–886.

56. Jiang, X.; Wang, Y.; Li, M. Selecting Water–Alcohol Mixed Solvent for Synthesis of Polydopamine Nano-Spheres Using Solubility Parameter. *Sci. Rep.* **2014**, *4*, 6070.

57. Rasool, M.A.; Vankelecom, I.F.J. Use of $\gamma$-Valerolactone and Glycerol Derivatives as Bio-Based Renewable Solvents for Membrane Preparation. *Green Chem.* **2019**, *21*, 1054–1064.

58. Yeow, M.L.; Liu, Y.T.; Li, K. Isothermal Phase Diagrams and Phase-Inversion Behavior of Poly(vinylidene fluoride)/Solvents/Additives/Water Systems. *J. Appl. Polym. Sci.* **2003**, *90*, 2150–2155.

59. Liu, F.; Hashim, N.A.; Liu, Y.; Abed, M.R.M.; Li, K. Progress in the Production and Modification of PVDF Membranes. *J. Membr. Sci.* **2011**, *375*, 1–27.

60. Fionah, A.; Hackett, C.; Aljewari, H.; Brady, L.; Alqhtani, F.; Escobar, I.C.; Thompson, A.K. Microcystin-LR Removal from Water via Enzymatic Linearization and Ultrafiltration. *Toxins* **2022**, *14*, 231.
